# Continuous glucose monitoring-derived time in range and CV are associated with altered tissue characteristics of the carotid artery wall in people with type 2 diabetes

**DOI:** 10.1007/s00125-023-06013-3

**Published:** 2023-09-26

**Authors:** Tomoya Mita, Naoto Katakami, Yosuke Okada, Hidenori Yoshii, Takeshi Osonoi, Keiko Nishida, Toshihiko Shiraiwa, Akira Kurozumi, Naohiro Taya, Satomi Wakasugi, Fumiya Sato, Ryota Ishii, Masahiko Gosho, Iichiro Shimomura, Hirotaka Watada

**Affiliations:** 1 Department of Metabolism & Endocrinology, Juntendo University Graduate School of Medicine, Tokyo, Japan; 2 Department of Metabolic Medicine, Osaka University Graduate School of Medicine, Osaka, Japan; 3 First Department of Internal Medicine, School of Medicine, University of Occupational and Environmental Health, Kitakyushu, Japan; 4 Department of Medicine, Diabetology & Endocrinology, Juntendo Tokyo Koto Geriatric Medical Center, Tokyo, Japan; 5 Nakakinen Clinic, Ibaraki City, Japan; 6 Nishida Keiko Diabetes Clinic, Kitakyushu, Japan; 7 Shiraiwa Medical Clinic, Osaka, Japan; 8 Department of Biostatistics, Faculty of Medicine, University of Tsukuba, Ibaraki, Japan

**Keywords:** Atherosclerosis, Continuous glucose monitoring, Glucose variability, Grey-scale median, Intima–media thickness

## Abstract

**Aims/hypothesis:**

Previous studies have suggested that glucose variability may accelerate atherosclerosis progression in people with type 2 diabetes. Current guidelines recommend assessing glycaemic control using continuous glucose monitoring (CGM), which provides a comprehensive glycaemic profile to supplement HbA_1c_ measurement. However, the association between CGM-derived metrics and atherosclerosis progression is not entirely clear.

**Methods:**

This exploratory study used baseline data and data obtained after 104 weeks from an ongoing prospective, multicentre, observational study. Six hundred study participants with type 2 diabetes and no apparent history of symptomatic cardiovascular disease underwent CGM and ultrasonographic atherosclerosis measurements of the carotid arteries, including the intima–media thickness (IMT) and grey-scale median (GSM), at baseline and 104 weeks. Non-invasive ultrasonic tissue characterisation of the carotid artery wall or plaque using the GSM reflects vascular composition. Multivariate regression models were used to analyse the association between CGM-derived indices, mainly time in range (TIR) and CV, and changes in carotid atherosclerosis index values.

**Results:**

Over the 104-week study period, there were modest increases in mean IMT (from 0.759±0.153 to 0.773±0.152 mm, *p*<0.001) and thickened-lesion GSM (from 43.5±19.5 to 53.9±23.5 units, *p*<0.001), but no significant changes in common carotid artery maximum-IMT (from 1.109±0.442 to 1.116±0.469 mm, *p*=0.453) or mean GSM (from 48.7±19.3 to 49.8±20.8 units, *p*=0.092). In a linear regression model with adjustment for possible atherosclerotic risk factors, including HbA_1c_, TIR and CV at baseline were significantly associated with the annual change in mean GSM (regression coefficient per 10% increase in TIR 0.52; 95% CI 0.06, 0.98; Hochberg-adjusted *p* value 0.038; regression coefficient per 1% increase in CV −0.12; 95% CI −0.22, −0.02; Hochberg-adjusted *p* value 0.038). TIR and CV at baseline were also significantly associated with the annual change in thickened-lesion GSM (regression coefficient per 10% increase in TIR 0.95; 95% CI 0.12, 1.79; Hochberg-adjusted *p* value 0.038; regression coefficient per 1% increase in CV −0.19; 95% CI −0.36, −0.01; Hochberg-adjusted *p* value 0.038). Participants who achieved target CGM-derived metrics at baseline, as proposed by an international consensus, showed significant annual changes in mean GSM compared with those who did not (0.94±6.88 vs −0.21±6.19 units/year, *p*=0.007).

**Conclusions/interpretation:**

TIR and CV were significantly associated with changes in the tissue characteristics of the carotid artery wall.

**Trial registration:**

University Hospital Medical Information Network Clinical Trials Registry, number UMIN000032325

**Graphical Abstract:**

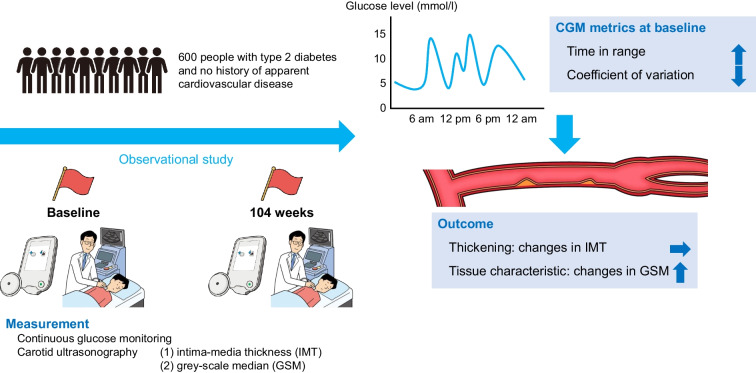

**Supplementary Information:**

The online version of this article (10.1007/s00125-023-06013-3) contains peer-reviewed but unedited supplementary material.


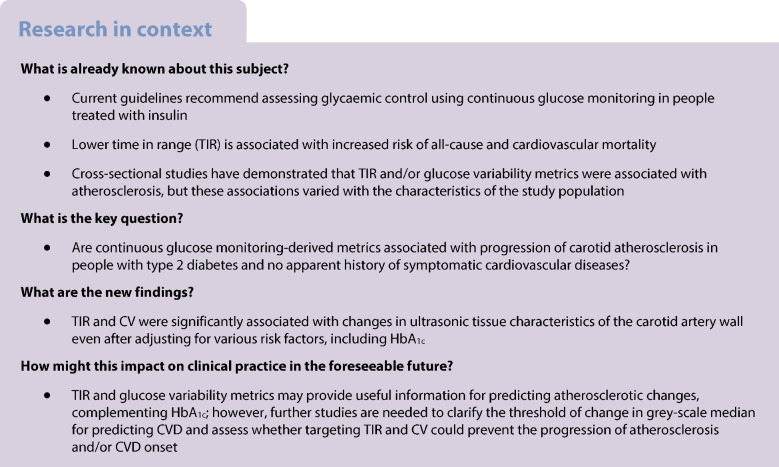


## Introduction

Type 2 diabetes is an independent risk factor for CVD, which is a major cause of death [1]. Given that the main cause of diabetic complications is damage to tissues and organs caused by persistent hyperglycaemia [2], achieving optimal glycaemic control is important to prevent the development and progression of CVD in people with this condition.

HbA_1c_ is the gold standard for assessing glycaemic control. Current guidelines developed by the ADA, the EASD and the Japan Diabetes Society have set an HbA_1c_ control target of less than 7% (53 mmol/mol) for optimal diabetes management [3–6]. However, the ADA and EASD guidelines specify that HbA_1c_ has a number of limitations [6, 7]. While some studies have demonstrated strong associations between HbA_1c_ levels and diabetic complications [8, 9], several large clinical trials have failed to show that intensive glycaemic control based on HbA_1c_ has beneficial effects on CVD onset in people with type 2 diabetes [10–12]. This is most likely because HbA_1c_ does not provide information on all glycaemic abnormalities that may play an important role in the development of CVD, such as glucose variability [13] and hypoglycaemia [14], although to our knowledge no prospective intervention studies have examined the relationship between glucose variability and CVD. Continuous glucose monitoring (CGM) has emerged as the optimal method to obtain a comprehensive glycaemic profile that includes such data. In particular, the consensus report by ADA and EASD recommended that assessments of glycaemic control include not only HbA_1c_ measurement but also time in range (TIR), defined as the percentage of the time spent within the target glucose range, because TIR is a useful metric of glycaemic control [6, 7].

Importantly, a recent cohort study in 6225 people with diabetes demonstrated that lower TIR assessed by CGM during hospitalisation was associated with an increased risk of all-cause and CVD mortality during a median follow-up of 6.9 years [15]. In addition, cross-sectional studies have demonstrated that TIR and/or glucose variability metrics are significantly associated with carotid artery intima–media thickening [16], the tissue characteristics of the carotid artery wall [17] and arterial stiffness [18, 19]. However, the association of TIR and glucose variability metrics with the progression of atherosclerosis has not yet been fully elucidated.

Carotid ultrasonography is a simple, non-invasive and inexpensive procedure for assessing the severity of atherosclerosis [20], while non-invasive ultrasonic tissue characterisation of the carotid artery wall or plaque using the grey-scale median (GSM) reflects vascular composition [21]. Lesions containing abundant lipids and haemorrhagic components are more echolucent (lower GSM), whereas stable components, including fibrous and calcified tissue, are more echogenic (higher GSM). A previous study demonstrated that people with type 2 diabetes had more echolucent plaques than those without the condition [22]. In addition, a recent study indicated that people with type 2 diabetes were at higher risk of CVD if they had low GSM plaques than if they did not [23]. Also, we recently showed that the change in mean GSM over time was useful for predicting CVD events in people with type 2 diabetes [24].

In this exploratory study, we investigated the relationship of TIR and glucose variability metrics with changes in carotid atherosclerosis, including intime–media thickness (IMT) and the tissue characteristics of the carotid artery wall, in people with type 2 diabetes with no apparent history of symptomatic CVD.

## Methods

### Study design

This study was an exploratory sub-analysis of an ongoing observational, prospective cohort study investigating the relationships between glucose fluctuations evaluated using CGM and the incidence of composite cardiovascular events over a 5-year follow-up period [25]. The primary aim of this study was to assess the relationship between TIR and CV at baseline, and changes in IMT and GSM over 104 weeks, with adjustment for conventional risk factors. The secondary aim was to assess the association of other CGM-derived metrics and HbA_1c_ with changes in IMT and GSM. This study used cohort study data obtained at baseline and at 104 weeks. This study has been registered in the University Hospital Medical Information Network Clinical Trials Registry, which is a non-profit organisation in Japan that meets the requirements of the International Committee of Medical Journal Editors (UMIN000032325).

### Study population

The study population consisted of Japanese people with type 2 diabetes who regularly attend the outpatient diabetes clinics of 34 institutions across Japan. The study design, inclusion criteria and exclusion criteria have been published previously [25] and are described in electronic supplementary material (ESM) Methods. Briefly, the study enrolled outpatients aged ≥30 years and ≤80 years who were on a stable glucose-lowering treatment regimen, defined as no changes in glucose-lowering medications (including new prescriptions) for 6 months before written informed consent was obtained, and with no anticipated changes in glucose-lowering medication from the time of enrolment until the application of CGM sensors. Insulin dose changes were allowed. People with a history of symptomatic cardiovascular events were excluded. Information on how representative this study sample was of the larger population of interest in terms of age, sex, ethnicity, regional and socioeconomic factors was not available. Sex and race/ethnicity were self-reported.

Consecutive people attending the clinic were screened. People who met the eligibility criteria were asked to participate in the present study. A total of 1000 people who met the eligibility criteria were recruited between May 2018 and March 2019. One person withdrew consent. Among the 999 participants, 600 for whom baseline carotid ultrasound images were obtained were included in this analysis. The protocol was approved by the institutional review board of each participating institution in compliance with the Declaration of Helsinki and current legal regulations in Japan. Written informed consent was obtained from all participants after a full explanation of the study.

### Biochemical tests

Blood samples were obtained after overnight fasting. Renal function, lipid levels and HbA_1c_ (standardised according to the National Glycohaemoglobin Standardisation Program) were measured using standard techniques. Urinary albumin excretion was measured by a latex agglutination assay using a spot urine sample. The eGFR was calculated using a previously defined formula [26].

### Continuous glucose monitoring using the FreeStyle Libre Pro

The FreeStyle Libre Pro CGM (FLP-CGM) device (Abbott Japan), which measures glucose levels every 15 min for up to 14 days, was used at baseline as previously reported [18, 25] and at 104 weeks. Other than wearing the FLP-CGM, there were no restrictions on participants’ daily lives. Downloaded datasets were analysed. Glucose variability was assessed based on the glucose CV [7]. CV (%) was calculated by dividing the SD by the mean of the corresponding glucose readings. TIR was defined as the percentage of time within the target range of 3.9–10.0 mmol/l. Time above range (TAR) >10 and >13.9 mmol/l (TAR^>10 mmol/l^ and TAR^>13.9 mmol/l^) were defined as the percentages of time above the corresponding blood glucose levels. Time below range (TBR) <3.9 and <3.0 mmol/l (TBR^<3.9 mmol/l^ and TBR^<3.0 mmol/l^) were defined as the percentages of time below the corresponding blood glucose levels [7]. Mean glucose, CV, TIR, TAR and TBR are among the primary measurable outcomes of CGM [27]. As a previous study demonstrated that FLP-CGM was less accurate during the first 24 h after insertion (from the first day to the second day) and during the last 4 days of its 14-day lifetime [28], we analysed FLP-CGM data obtained over the middle 8-day period.

### Measurement of carotid IMT

Ultrasonographic scans of the carotid artery were performed by expert sonographers who had been specifically trained to perform the prescribed study examination using the same equipment in each setting, as reported previously [29, 30]. Briefly, the extracranial common carotid artery (CCA), the carotid bulb and the internal carotid artery in the neck were scanned bilaterally in transverse projections and at least three different longitudinal projections; in addition, the site of greatest thickness, including plaque lesions, was identified along the arterial walls. The IMT represents the distance between two parallel echogenic lines corresponding to the vascular lumen and the adventitia. To avoid inter-reader variability, all scans were electronically stored and emailed to the central office (IMT Evaluation Committee, Osaka, Japan) to be read in random order by a single experienced reader who was blinded to the clinical characteristics of the participants, using Intimascope automated digital edge-detection software (MediaCross, Japan) [29, 30]. The software system calculates the mean of approximately 200 IMT values in the segment that is 2 cm proximal to the dilation of the carotid bulb (mean IMT-CCA), and CCA-max-IMT was defined as the higher of the right and left values.

**Fig. 1:**
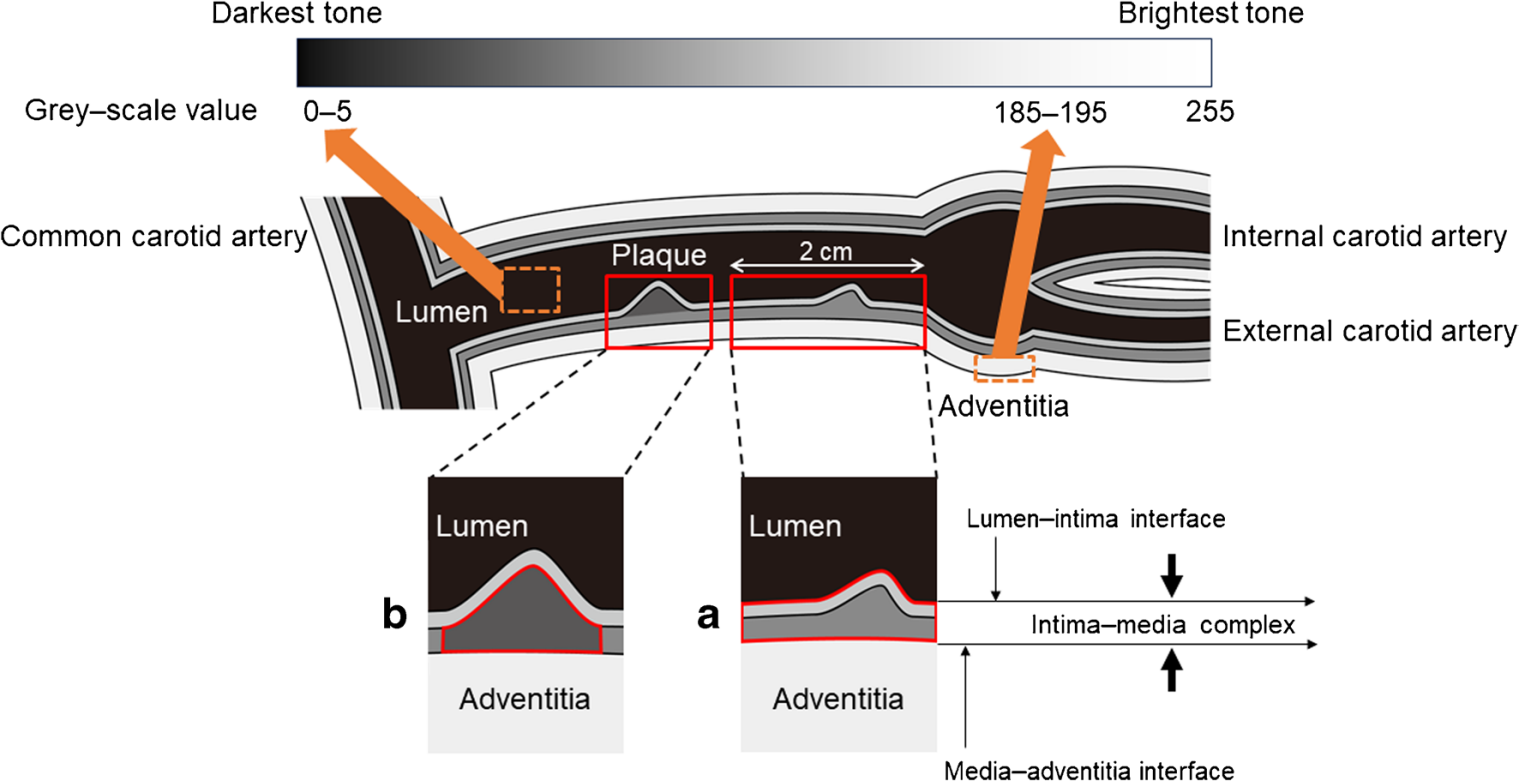
Methods of GSM evaluation. Standardisation of the B-mode image was performed using a curve option, so that the GSM for blood ranged from 0 to 5 and that for the adventitia ranged from 185 to 195. (**a**) The right and left mean IMT areas (intima–media complex of the segment 2 cm proximal to dilation of the carotid bulb) were then delineated using a freehand tool (red frame), and the GSM of the selected area was read from the entire delineated area. The mean GSM was defined as the average of the right and left values. (**b**) If there were atherosclerotic thickened lesions (focal IMT ≥1.0 mm), the GSM of these lesions was measured using the same method in the right and left arteries. We defined these atherosclerotic thickened lesions as ‘carotid plaque’. The lesions were delineated using a freehand tool (red frame), and the GSM value of each carotid plaque was read from the entire delineated area. If multiple thickened lesions were found in the same individual, the lesion with the greatest thickness was subjected to GSM measurement separately in the left and right carotids, and then the lower of the right and left values, defined as ‘thickened-lesion GSM’, was used as the representative value

The echogenicity of the arterial wall was evaluated based on the GSM method, with a grey-scale range of 0–255 (where 0 is the darkest tone and 255 is the brightest), as described previously [17]. Adobe Photoshop software version 7.0 (Adobe Systems, USA) was used for image standardisation and calculation of grey-scale values. The methods used for GSM measurement are shown in detail in Fig. 1.

The annual change in each value was calculated as (final value − initial value)/observation period. The intra-reader CVs for mean IMT, CCA-max-IMT and GSM measurements were 2.0%, 2.7% and 2.9%, respectively, for 40 consecutively replicated measurements [17].

### Statistical analysis

The results are presented as mean ± SD or median (IQR) for continuous variables, and as number (proportion) of patients for categorical variables. Changes from baseline to 104 weeks were assessed using the one-sample *t* test and Wilcoxon’s signed-rank test for continuous variables, and McNemar’s test for categorical variables. A pre-specified multivariate linear regression analysis was performed to investigate whether FLP-CGM-derived metrics were associated with changes in index values for carotid atherosclerosis, which included mean IMT-CCA, CCA-max-IMT, mean GSM and thickened-lesion GSM. The model included the following pre-specified conventional risk factors [18, 31]: age, sex, index values for carotid atherosclerosis, BMI, duration of diabetes, HbA_1c_, systolic BP, total cholesterol, HDL-cholesterol, triacylglycerols, uric acid, eGFR, urinary albumin excretion, presence of diabetic retinopathy, smoking status, alcohol consumption, use of insulin therapy, use of angiotensin-converting enzyme inhibitors and/or angiotensin II receptor blockers, use of statins and use of anti-platelet agents at baseline [32–37]. The Hochberg method was used to control multiple testing [38] as a post hoc analysis.

Next, we performed a pre-specified sensitivity analysis to investigate the relationship between FLP-CGM-derived metrics and the progression of carotid atherosclerosis. Participants were divided into three groups based on tertiles of FLP-CGM-derived metrics at baseline. Trend association across these groups was evaluated by linear regression analysis adjusted for age, sex and each index value for carotid atherosclerosis at baseline.

Finally, we performed a post hoc sensitivity analysis to investigate how atherosclerosis progression was affected by achieving target CGM-derived metrics proposed by an international consensus [27]. To do this, we classified participants into two groups: those who did or did not achieve all target CGM-derived metrics at baseline: TIR >70%, TAR^>10 mmol/l^ <25%, TAR^>13.9 mmol/l^ <5%, TBR^<3.9 mmol/l^ <4% and TBR^<3.0 mmol/l^ <1%. Comparison between the two groups was performed using a linear regression model adjusted for potential conventional risk factors for atherosclerosis.

All statistical tests were two-sided with a 5% significance level. All analyses were performed using SAS software version 9.4 (SAS Institute, USA).

## Results

### Participants’ demographic and background characteristics, and changes in clinical parameters during the study period

The data of 600 participants with type 2 diabetes were available at baseline. Forty-seven participants did not undergo carotid ultrasonography at 104 weeks: 35 were lost to follow-up and 12 refused to undergo the examination. The baseline clinical characteristics of the 600 participants are summarised in Table 1. The mean age was 64.9±9.2 years, 63.2% were male, and the mean HbA_1c_ was 7.0±0.8% (53.5±9.0 mmol/mol). Over 104 weeks of follow-up, HbA_1c_ was not significantly changed, and there was a modest but significant increase in CV. With respect to other conventional atherosclerotic risk factors, BMI was slightly decreased, and renal function was slightly deteriorated, as determined by eGFR. The frequency of use of oral glucose-lowering agents, anti-hypertensive agents, statins and anti-platelet agents increased significantly during the 104-week study period.

**Table 1:** Participant demographic and background characteristics during the study period

Variable	Baseline	104 weeks	*p* value
Age (years)	64.9±9.2 (*n*=600)		
Male (%)	379 (63.2)		
Estimated duration of diabetes (years)	11.0 (6.0–18.0)		
Ever smoker (%)	327 (54.5)		
BMI (kg/m^2^)	24.6±3.8 (*n*=600)	24.5±3.8 (*n*=566)	0.028*
Systolic BP (mmHg)	132.0±14.8 (*n*=600)	132.5±15.7 (*n*=566)	0.590
Diastolic BP (mmHg)	76.4±10.9 (*n*=600)	76.2±11.2 (*n*=566)	0.425
HbA_1c_ (%)	7.0±0.8 (*n*=600)	7.1±0.8 (*n*=565)	0.090
HbA_1c_ (mmol/mol)	53.5±9.0 (*n*=600)	54.1±9.1 (*n*=565)	0.090
Total cholesterol (mmol/l)	4.77±0.81 (*n*=578)	4.72±0.80 (*n*=553)	0.183
LDL-cholesterol (mmol/l)	2.65±0.67 (*n*=596)	2.59±0.68 (*n*=562)	0.063
HDL-cholesterol (mmol/l)	1.55±0.41 (*n*=599)	1.56±0.42 (*n*=566)	0.098
Triacylglycerols (mmol/l)	1.12 (0.81–1.56) (*n*=600)	1.13 (0.80–1.59) (*n*=565)	0.622
Uric acid (µmol/l)	308.4±73.5 (*n*=598)	305.6±73.1 (*n*=562)	0.117
eGFR (ml/min per 1.73 m^2^)	70.0±18.2 (*n*=600)	68.7±18.2 (*n*=565)	<0.001*
Urinary albumin excretion (mg/mmol)	1.6 (0.7–4.6) (*n*=588)	1.5 (0.7–5.1) (*n*=544)	0.002*
Use of medications (%)			
Oral glucose-lowering agents	550 (91.7) (*n*=600)	536 (94.5) (*n*=567)	<0.001*
Metformin	337 (56.2) (*n*=600)	351 (61.9) (*n*=567)	<0.001*
Sulfonylureas	69 (11.5) (*n*=600)	58 (10.2) (*n*=567)	0.059
Glinides	38 (6.3) (*n*=600)	65 (11.5) (*n*=567)	<0.001*
Dipeptidyl peptidase-4 inhibitors	342 (57.0) (*n*=600)	325 (57.3) (*n*=567)	0.889
Sodium-glucose cotransporter 2 inhibitors	149 (24.8) (*n*=600)	170 (30.0) (*n*=567)	<0.001*
Thiazolidinediones	94 (15.7) (*n*=600)	89 (15.7) (*n*=567)	0.197
α-glucosidase inhibitors	131 (21.8) (*n*=600)	142 (25.0) (*n*=567)	0.002*
Glucagon-like peptide-1 antagonists	42 (7.0) (*n*=600)	61 (10.8) (*n*=567)	<0.001*
Insulin	97 (16.2) (*n*=600)	100 (17.6) (*n*=567)	0.117
Angiotensin-converting enzyme inhibitors	12 (2.0) (*n*=600)	12 (2.1) (*n*=567)	1.000
Angiotensin II receptor blockers	256 (42.7) (*n*=600)	262 (46.2) (*n*=567)	0.008*
Calcium channel blockers	164 (27.3) (*n*=600)	179 (31.6) (*n*=567)	0.002*
Statins	320 (53.3) (*n*=600)	329 (58.0) (*n*=567)	<0.001*
Anti-platelet agents	25 (4.2) (*n*=600)	32 (5.6) (*n*=567)	0.020*
FLP-CGM-derived metrics			
Mean glucose (mmol/l)	7.62±1.68 (*n*=600)	7.64±1.47 (*n*=530)	0.923
CV (%)	25.8±5.9 (*n*=600)	26.6±5.8 (*n*=530)	<0.001*
TIR (%)	80.8±17.8 (*n*=600)	80.7±15.6 (*n*=530)	0.734
TAR^>10 mmol/l^ (%)	17.0±18.2 (*n*=600)	17.2±16.0 (*n*=530)	0.838
TAR^>13.9 mmol/l^ (%)	3.1±8.0 (*n*=600)	2.7±6.3 (*n*=530)	0.147
TBR^<3.9 mmol/l^ (%)	2.2±4.7 (*n*=600)	2.1±4.9 (*n*=530)	0.714
TBR^<3.0 mmol/l^ (%)	0.3±1.4 (*n*=600)	0.3±1.4 (*n*=530)	0.822
Index values for carotid atherosclerosis			
Mean IMT (mm)	0.759±0.153 (*n*=600)	0.773±0.152 (*n*=552)	<0.001*
Annual change in mean IMT (mm/year)	0.008±0.046		
CCA-max-IMT (mm)	1.109±0.442 (*n*=600)	1.116±0.469 (*n*=552)	0.453
Annual change in CCA-max-IMT (mm/year)	0.004±0.128		
Mean GSM (units)	48.7±19.3 (*n*=599)	49.8±20.8 (*n*=552)	0.092
Annual change in mean GSM (units/year)	0.48±6.63		
Thickened-lesion GSM (units)	43.5±19.5 (*n*=566)	53.9±23.5 (*n*=505)	<0.001*
Annual change in thickened-lesion GSM (units/year)	5.3±11.6		

Data are means ± SD, median (IQR) or number of patients (%)

Asterisks indicate variables that are statistically significantly different between baseline and 104 weeks

A modest but significant increase in mean IMT was observed over 104 weeks, but there was no significant change in CCA-max-IMT. The annual changes in mean IMT and CCA-max-IMT were 0.008±0.046 and 0.004±0.128 mm/year, respectively. For GSM, a modest but significant increase in thickened-lesion GSM was observed over 104 weeks, but there was no significant change in mean GSM. The annual changes in mean GSM and thickened-lesion GSM were 0.48±6.63 and 5.3±11.6 units/year, respectively.

### Relationship between CV and TIR at baseline, and changes in IMT and GSM

The primary aim of this study was to use multivariate linear regression analysis to assess the relationship between CV and TIR at baseline, and changes in IMT and GSM over 104 weeks. CV and TIR were not associated with the annual changes in mean IMT or CCA-max-IMT (Table 2). However, CV and TIR were significantly associated with the annual changes in mean GSM and thickened-lesion GSM (Table 3). As CV at baseline increased by 1%, mean GSM decreased by 0.12 units/year. As TIR at baseline increased by 10%, mean GSM increased by 0.52 units/year. Similarly, as CV at baseline increased by 1%, thickened-lesion GSM decreased by 0.19 units/year. As TIR at baseline increased by 10%, thickened-lesion GSM increased by 0.95 units/year.

**Table 2:** Associations between CV or TIR and annual changes in IMT in the adjusted model

Variable	Regression coefficient (95% CI)	Crude *p* value	Hochberg-adjusted *p* value
Mean IMT change (*n*=518)			
CV (%) (1% increase)	−0.000 (−0.001, 0.001)	0.602	0.855
TIR (10% increase)	0.000 (−0.003, 0.004)	0.810	0.855
CCA-max-IMT change (*n*=518)			
CV (%) (1% increase)	0.000 (−0.002, 0.002)	0.855	0.855
TIR (10% increase)	−0.002 (−0.011, 0.007)	0.671	0.855

The model included age, sex and index values for carotid atherosclerosis at baseline, BMI, duration of diabetes, HbA_1c_, systolic BP, total cholesterol, HDL-cholesterol, logarithm of triacylglycerols, serum uric acid, eGFR, logarithm of urinary albumin excretion, presence of diabetic retinopathy, smoking status (never smoker, previous smoker, or current smoker), alcohol consumption, use of insulin therapy, use of angiotensin-converting enzyme inhibitors and/or angiotensin II receptor blockers, use of statins and use of anti-platelet agents. The presence of diabetic retinopathy (including simple diabetic retinopathy, pre-proliferative diabetic retinopathy and proliferative diabetic retinopathy) was determined based on medical records

**Table 3:** Associations between CV or TIR and annual changes in GSM in the adjusted model

Variable	Regression coefficient (95% CI)	Crude *p* value	Hochberg-adjusted *p* value
Mean GSM change (*n*=517)			
CV (%) (1% increase)	−0.12 (−0.22, −0.02)	0.016	0.038
TIR (10% increase)	0.52 (0.06, 0.98)	0.027	0.038
Thickened-lesion GSM change (*n*=456)			
CV (%) (1% increase)	−0.19 (−0.36, −0.01)	0.038	0.038
TIR (10% increase)	0.95 (0.12, 1.79)	0.025	0.038

Potential risk factors as listed in the footnote to Table 2 were included in the model

### Association of FLP-CGM-derived metrics and HbA_1c_ at baseline with changes in IMT and GSM

The secondary aim of this study was to use multivariate linear regression analysis to assess the relationship between other FLP-CGM-derived metrics, which included mean glucose, TAR^>10 mmol/l^, TBR^<3.9 mmol/l^ and HbA_1c_ at baseline, and changes in IMT and GSM over 104 weeks. Neither FLP-CGM-derived metrics nor HbA_1c_ were associated with changes in mean IMT, CCA-max-IMT, mean GSM or thickened-lesion GSM after adjusting for multiple testing (Table 4).

**Table 4:** Associations between FLP-CGM-derived metrics or HbA_1c_ and annual changes in index values for carotid atherosclerosis in the adjusted model

Variable	Regression coefficient (95% CI)	Crude *p* value	Hochberg-adjusted *p* value
Mean IMT change (*n*=518)			
Mean glucose (1 mmol/l increase)	−0.003 (−0.006, 0.001)	0.161	0.971
TAR^>10 mmol/l^ (1% increase)	0.000 (0.000, 0.000)	0.310	0.971
TBR^<3.9 mmol/l^ (1% increase)	0.001 (0.000, 0.002)	0.032	0.466
HbA_1c_ (1% increase) (excluding HbA_1c_)	−0.001 (−0.006, 0.004)	0.765	0.971
CCA-max-IMT change (*n*=518)			
Mean glucose (1 mmol/l increase)	0.000 (−0.010, 0.011)	0.971	0.971
TAR^>10 mmol/l^ (1% increase)	0.000 (−0.001, 0.001)	0.910	0.971
TBR^<3.9 mmol/l^ (1% increase)	0.001 (−0.001, 0.004)	0.369	0.971
HbA_1c_ (1% increase) (excluding HbA_1c_)	−0.004 (−0.018, 0.011)	0.635	0.971
Mean GSM change (*n*=517)			
Mean glucose (1 mmol/l increase)	−0.35 (−0.89, 0.18)	0.196	0.971
TAR^>10 mmol/l^ (1% increase)	−0.05 (−0.10, 0.00)	0.035	0.466
TBR^<3.9 mmol/l^ (1% increase)	−0.03 (−0.16, 0.10)	0.648	0.971
HbA_1c_ (1% increase) (excluding HbA_1c_)	−0.13 (−0.87, 0.62)	0.737	0.971
Thickened-lesion GSM change (*n*=456)			
Mean glucose (1 mmol/l increase)	−0.93 (−1.90, 0.05)	0.064	0.766
TAR^>10 mmol/l^ (1% increase)	−0.09 (−0.18, −0.01)	0.036	0.466
TBR^<3.9 mmol/l^ (1% increase)	−0.07 (−0.30, 0.17)	0.575	0.971
HbA_1c_ (1% increase) (excluding HbA_1c_)	−1.48 (−2.82, −0.14)	0.031	0.466

Potential risk factors as listed in the footnote to Table 2 were included in the model

### Relationship between FLP-CGM-derived metrics and changes in index values for carotid atherosclerosis

Next, we performed a pre-defined sensitivity analysis to investigate the relationship between FLP-CGM-derived metrics and the progression of carotid atherosclerosis. Participants were divided into three groups based on tertiles of FLP-CGM-derived metrics at baseline. There was no significant association between FLP-CGM-derived metrics classified by tertiles and the annual changes in mean IMT and CCA-max-IMT in a linear regression model adjusted for age, sex and each IMT value at baseline (ESM Table 1). In a linear regression model adjusted for age, sex and mean GSM at baseline, lower CV and higher TIR classified by tertiles were significantly associated with an annual increase in mean GSM (ESM Table 1). Also, in a linear regression model adjusted for age, sex and thickened-lesion GSM at baseline, lower mean glucose, CV, TAR^>13.9 mmol/l^ and higher TIR classified by tertiles were significantly associated with an annual increase in thickened-lesion GSM (ESM Table 1). However, no significant association was observed between HbA_1c_ values classified by tertile and the annual changes in all index values for carotid atherosclerosis (ESM Table 1).

### Relationship between changes in FLP-CGM-derived metrics and changes in index values for carotid atherosclerosis

We performed a pre-specified exploratory analysis to investigate the relationship between changes in FLP-CGM-derived metrics from baseline to 104 weeks and the progression of carotid atherosclerosis over 104 weeks. There were no significant relationships between changes in FLP-CGM-derived metrics and the annual changes in any of the index values for carotid atherosclerosis (ESM Table 2). In addition, in adjusted linear regression models, no significant association was observed between changes in FLP-CGM-derived metrics and the annual changes in these values (ESM Tables 3 and 4).

### Relationship between FLP-CGM-derived metrics and carotid atherosclerosis among groups based on achievement of target CGM-derived metrics

Finally, we performed a post hoc sensitivity analysis to investigate how atherosclerosis progression was affected by achieving target CGM-derived metrics. Participants were divided into two groups based on whether or not they achieved target CGM-derived metrics at baseline. There was a significant difference in annual change in mean GSM between participants who achieved the target CGM-derived metrics and those who did not after adjustment for other conventional potential risk factors for atherosclerosis (Table 5).

**Table 5:** Comparisons of annual changes in index values for carotid atherosclerosis between participants who did or did not achieve a TIR target

	Change	*p* for group comparison
Mean IMT change (mm/year) (*n*=552)		
Non-TIR target achievement group (*n*=222)	0.008±0.045	0.894
TIR target achievement group (*n*=330)	0.007±0.047	
CCA-max-IMT change (mm/year) (*n*=552)		
Non-TIR target achievement group (*n*=222)	0.009±0.155	0.668
TIR target achievement group (*n*=330)	0.001±0.107	
Mean GSM change (units/year) (*n*=551)		
Non-TIR target achievement group (*n*=220)	−0.21±6.19	0.007*
TIR target achievement group (*n*=331)	0.94±6.88	
Thickened-lesion GSM change (units/year) (*n*=486)		
Non-TIR target achievement group (*n*=198)	4.9±11.4	0.171
TIR target achievement group (*n*=288)	5.7±11.8	

The TIR target achievement group consisted of participants who fulfilled all of the following criteria at baseline: TIR >70%, TAR^>10 mmol/l^ <25%, TAR^>13.9 mmol/l^ <5%, TBR^<3.9 mmol/l^ <4% and TBR^<3.0 mmol/l^ <1%. Group comparisons were performed using a linear regression model adjusted for potential conventional risk factors for atherosclerosis.

Asterisk indicates a statistically significantly difference between those who achieved the target and those who did not

## Discussion

To our knowledge, this is the first study to reveal that TIR and CV are associated with changes in ultrasonic tissue characteristics of the carotid artery wall, even after adjusting for various risk factors, including HbA_1c_. In addition, participants who achieved the target CGM-derived metrics proposed by the international consensus demonstrated significant annual changes in mean GSM compared with those who did not. These results underscore the importance of including these CGM-derived metrics as a part of optimal diabetes management in conjunction with HbA_1c_, and indicate the need for further studies to clarify the threshold of change in GSM for predicting CVD and to assess whether targeting TIR and CV could prevent the progression of atherosclerosis and/or CVD onset.

A previous study demonstrated that every 10 unit/year increment in mean GSM was associated with an approximately 73% risk reduction in CVD [24]. Accordingly, the increase in mean GSM of approximately 1 unit/year found in participants with lower CV and higher TIR classified by tertiles, and those who achieved the target CGM metrics may be clinically relevant. Thus, achieving glycaemic control using CGM may be beneficial for reducing the risk of CVD. However, observing changes in thickened-lesion GSM is also useful for evaluating the progression of atherosclerosis. Notably, we found that there was a significant greater change in thickened-lesion GSM in participants with lower CV and higher TIR classified by tertiles, and thickened-lesion GSM change was numerically but non-significantly greater in participants who achieved the target CGM metrics than in those who did not. Theoretically, assessment of thickened lesions in the carotid arteries, including plaque, is more useful for predicting future CVD than the evaluation of mean GSM. However, it remains largely unknown whether longitudinal changes in thickened-lesion GSM are associated with the risk of CVD. This may be because the thickened carotid lesions evaluated at baseline were not necessarily always the same as those evaluated at other follow-up points, because of the inability to track changes in individual thickened lesions over time. At present, it is reasonable to state that changes in mean GSM constitute a more reproducible and reliable marker for predicting CVD.

Previous cross-sectional studies demonstrated that glucose variability was associated with coronary or carotid plaque tissue characteristics [17, 39, 40]. In addition, to our knowledge, the current study is the first to provide evidence that CV was associated with changes in ultrasonic tissue characteristics of the carotid artery wall. Furthermore, the relationship between TIR, which may comprehensively reflect both glycaemic level and glycaemic variability [41], and mean GSM change or thickened-lesion GSM change, did not reach significance after adjusting for CV (data not shown), suggesting that glycaemic variability may be a major contributor. Although the exact mechanism of how glucose variability contributes to the tissue characteristics of the carotid vascular wall remains unclear, we propose the following possible scenario. Previous studies have shown that glucose variability induces inflammation and increases oxidative stress to a greater extent than chronic persistent hyperglycaemia [42], thus contributing to vascular damage. In agreement with these data, endothelial cell apoptosis caused by oxidative stress was more pronounced after intermittent hyperglycaemia than after persistent hyperglycaemia [43]. Accordingly, vascular walls may be damaged more by glucose variability than by chronic persistent hyperglycaemia.

In cross-sectional studies, the relationship between glucose variability and IMT is inconsistent [16, 17, 19]. In addition, we found no significant association between FLP-CGM-derived metrics, including glucose variability metrics, and annual changes in mean IMT and CCA-max-IMT. Thus, it remains inconclusive whether glucose variability affects carotid IMT. Previous studies demonstrated that people with type 2 diabetes had a greater mean IMT than those without [44, 45], and their annual increase in IMT (0.034 mm/year) was also greater (0.007–0.008 mm/year in a healthy population) [33]. Our study enrolled people with a stable glucose-lowering treatment regimen and no apparent history of symptomatic CVD who visited an outpatient clinic for routine medical care. Therefore, our participants had relatively low HbA_1c_ levels as well as higher TIR and lower CV. Also, BP and lipid variables were relatively well controlled, and BMI was not very high. Not surprisingly, the mean IMT of 0.759 mm in our participants was relatively low, and the annual changes in mean IMT (0.008 mm/year) and CCA-max-IMT (0.004 mm/year) were smaller than those in a previous prospective cohort study [46]. Under these conditions, glucose variability may not greatly contribute to the progression of carotid IMT. Further studies are needed to clarify these relationships.

It remains unclear why FLP-CGM-derived metrics were associated with the annual changes in GSM but not the changes in IMT. This may be because IMT and GSM have different relationships with major atherosclerosis risk factors [32, 47]. Furthermore, carotid IMT and GSM reflect different pathogenic processes of atherosclerosis, even though the two variables are modestly correlated with each other [32]. A previous study examining the morphological features of early human atherosclerosis demonstrated that, in atherosclerosis-prone arteries, including carotid arteries, extracellular lipid deposition begins to occur in the outer layer of pre-existing lesions with diffuse intimal thickening [48]. As the lesion progresses, lipids continue to accumulate in the outer layer of the thickened intima of the fatty streak, but do not cause a biologically important change in intimal thickness [49]. Then, stimulated macrophages accumulate in the fatty streak and infiltrate the accumulated lipid, leading to pathological intimal thickening with foam cells [49]. Accordingly, a change in tissue characteristics can precede IMT thickening in the early stage of atherosclerosis. Taken together, it is possible that persistent hyperglycaemia, glucose fluctuations and/or hypoglycaemia differentially affect IMT thickening and tissue characteristics of the vascular wall.

The strengths of this study included its relatively large sample size and multicentre design. However, the study had several limitations. First, it was a short-term, observational study, and therefore had drawbacks similar to all analyses of this type. Second, FLP-CGM-derived metrics were evaluated based on FLP-CGM measurements obtained during limited periods of 8 consecutive days at baseline and at 104 weeks. Thus, these metrics may not represent the participants’ overall glycaemic control. To most accurately assess baseline glucose variability using FLP-CGM, we only recruited people who were on a stable glucose-lowering treatment regimen. In addition, we employed a blind CGM system that prevented participants from altering their lifestyle behaviours based on the results of glucose readings. However, FLP-CGM measurements at 104 weeks were not performed in all participants and were obtained in the absence of any treatment restrictions. Last-minute changes in medications may greatly affect FLP-CGM metrics. This limitation may have made it difficult to evaluate the relationships between changes in FLP-CGM metrics and changes in index values for carotid atherosclerosis. Similarly, post hoc analysis showed no significant associations between mean values in FLP-CGM-derived metrics at baseline and 104 weeks and the annual changes in these values (data not shown). Repeated FLP-CGM measurements during the study period would have been required to clarify this point. Third, potential confounders were not included in the multivariate regression analysis. In particular, changes in the frequency of medication use, including use of glucagon-like peptide-1 antagonists, may have affected the results. Fourth, we recruited Japanese people with type 2 diabetes and without a history of symptomatic CVD. Given these inclusion criteria, a very limited number of people with carotid artery stenosis were included, although approximately 80% of participants had one or more carotid plaques at baseline. In addition, our participants had a TIR of approximately 80% and a CV of approximately 26% at baseline and 104 weeks. Accordingly, in our participants, TIR was more than acceptable and CV was much lower than the target CGM-derived metrics proposed by an international consensus [27], suggesting that blood glucose in our participants was relatively well controlled. These constraints may have limited the generalisability of our results. Finally, multiple testing in exploratory and sensitivity analyses increases the chance of false-positive findings, and thus our results should be interpreted with caution.

In conclusion, TIR and CV were significantly associated with changes in the tissue characteristics of the carotid artery wall, independently of HbA_1c_, in people with type 2 diabetes and no apparent history of symptomatic CVD. Thus, in conjunction with HbA_1c_, these metrics may provide useful information for predicting atherosclerotic changes.

### Supplementary Information

Below is the link to the electronic supplementary material.

Supplementary file1 (PDF 237 KB)

## Data Availability

All data generated or analysed during this study are included in this article or its supplementary material files. Further enquiries may be directed to the corresponding author.

## Abbreviations

CCA: Common carotid artery
CGM: Continuous glucose monitoring
FLP-CGM: FreeStyle Libre Pro continuous glucose monitoring
GSM: Grey-scale median
IMT: Intima–media thickness
TAR: Time above range
TBR: Time below range
TIR: Time in range
