# Supplementary material for: Continuous glucose monitoring-derived time in range and CV are associated with altered tissue characteristics of the carotid artery wall in people with type 2 diabetes
Source: Diabetologia. 2023 Sep 26;66(12):2356–67. doi: 10.1007/s00125-023-06013-3 (PMC10627957; doi:10.1007/s00125-023-06013-3)
Supplement: Supplementary file 1 — Supplementary file1 (PDF 237 KB) [file 125_2023_6013_MOESM1_ESM.pdf]

**SUPPLEMENTAL MATERIAL**

**ESM Methods.**

Inclusion criteria:

1) ≥30 years of age and ≤80 years of age (regardless of sex)

2) receiving treatment for type 2 diabetes at one of the above outpatient clinics

3) signing a consent form for participation in the study

4) no changes in glucose-lowering medications (including new prescriptions) for 6 months before written informed consent was obtained, and with no anticipated changes in glucose-lowering medication from the time of enrolment until the application of CGM sensors. Insulin dose changes were allowed.

Exclusion criteria:

1) type 1 or secondary diabetes

2) presence of severe infectious disease pre- or postoperatively or severe trauma

3) history of myocardial infarction, angina pectoris, cerebral stroke, cerebral infarction, or arteriosclerosis obliterans

4) current treatment with artificial dialysis

5) moderate liver dysfunction (aspartate aminotransferase ≥100 IU/l)

6) moderate or severe heart failure (New York Heart Association stage III or worse)

7) pregnancy, lactation, possible pregnancy, or plans to become pregnant during the study period

8) present or past history of a malignant tumor (however, patients not currently receiving medication for a malignant tumor, with no disease recurrence to date, and without recurrence risks during this study are allowed to participate)

9) use of a sensor-augmented insulin pump

10) type 2 diabetes diagnosis within the past year

11) judged as ineligible by the clinical investigators

**ESM Table 1. Comparisons of Annual Changes in Index Values of Carotid Atherosclerosis between Tertiles of FLP-CGM–derived Metrics**

| | Bottom tertile | Middle tertile | Top tertile | *P* for trend |
|---|---|---|---|---|
| **Mean glucose (mmol/L)** | **≤6.801** | **6.802 to 7.903** | **>7.903** | |
| Mean IMT change (mm/year) (*n*=522) | 0.012 ± 0.048 | 0.006 ± 0.047 | 0.004 ± 0.042 | 0.143 |
| CCA-max-IMT change (mm/year) (*n*=552) | 0.017 ± 0.147 | -0.008 ± 0.108 | 0.003 ± 0.127 | 0.421 |
| Mean GSM change(units/year) (*n*=551) | 0.51 ± 6.75 | 0.60 ± 6.81 | 0.32 ± 6.36 | 0.282 |
| Thickened-lesion GSM change (units/year) (*n*=486) | 6.1 ± 12.9 | 5.2 ± 10.8 | 4.7 ± 11.2 | **0.010** |
| | | | | |
| **CV (mmol/L)** | **≤22.8** | **22.9 to 27.7** | **>27.7** | |
| Mean IMT change (mm/year) (*n*=522) | 0.008 ± 0.038 | 0.009 ± 0.051 | 0.006 ± 0.049 | 0.528 |
| CCA-max-IMT change (mm/year) (*n*=552) | -0.001 ± 0.110 | 0.009 ± 0.152 | 0.005 ± 0.121 | 0.599 |
| Mean GSM change(units/year) (*n*=551) | 1.35 ± 6.21 | 0.28 ± 6.76 | -0.23 ± 6.85 | **0.003** |
| Thickened-lesion GSM (units/year) (*n*=486) | 6.6 ± 12.3 | 5.0 ± 12.1 | 4.4 ± 10.2 | **0.002** |
| | | | | |
| **TIR (%)** | **≤79.95** | **79.96 to 90.76** | **>90.76** | |
| Mean IMT change (mm/year) (*n*=552) | 0.009 ± 0.044 | 0.004 ± 0.048 | 0.010 ± 0.046 | 0.821 |
| CCA-max-IMT change (mm/year) (*n*=552) | 0.006 ± 0.116 | 0.000 ± 0.128 | 0.006 ± 0.140 | 0.808 |
| Mean GSM change(units/year) (*n*=551) | 0.34 ± 6.33 | 0.07 ± 6.95 | 1.01 ± 6.60 | **0.043** |
| Thickened-lesion GSM change (units/year) (*n*=486) | 4.8 ± 10.4 | 5.4 ± 11.3 | 5.9 ± 13.1 | **0.001** |
| | | | | |
| **TAR$^{>10\ mmol/L}$ (%)** | **≤5.47** | **5.48 to 17.71** | **>17.71** | |
| Mean IMT change (mm/year) (*n*=552) | 0.009 ± 0.044 | 0.007 ± 0.051 | 0.007 ± 0.043 | 0.407 |
| CCA-max-IMT change (mm/year) (*n*=552) | 0.009 ± 0.145 | 0.001 ± 0.112 | 0.002 ± 0.125 | 0.597 |
| Mean GSM change(units/year) (*n*=551) | 0.53 ± 6.61 | 0.62 ± 6.97 | 0.29 ± 6.35 | 0.127 |
| Thickened lesion-GSM change (units/year) (*n*=486) | 6.1 ± 13.0 | 4.9 ± 11.2 | 5.0 ± 10.5 | **0.003** |
| | | | | |
| **TBR$^{<3.9\ mmol/L}$ (%)** | **≤0.00** | **0.01 to 1.04** | **>1.04** | |
| Mean IMT change (mm/year) (*n*=552) | 0.008 ± 0.049 | 0.007 ± 0.036 | 0.007 ± 0.047 | 0.777 |

| | | | | |
|---|---|---|---|---|
| CCA-max-IMT change (mm/year) (*n*=552) | 0.002 ± 0.122 | 0.001 ± 0.089 | 0.009 ± 0.152 | 0.764 |
| Mean GSM change(units/year) (*n*=551) | 0.65 ± 6.36 | 1.23 ± 7.50 | -0.17 ± 6.42 | 0.513 |
| Thickened-lesion GSM change (units/year) (*n*=486) | 5.2 ± 11.0 | 4.6 ± 11.4 | 6.0 ± 12.5 | 0.256 |
| | | | | |
| **$HbA_{1c}$ (%) (mmol/mol)** | **≤6.6** | **6.7 to 7.2** | **>7.2** | |
| Mean IMT change (mm/year) (*n*=552) | 0.009 ± 0.051 | 0.005 ± 0.041 | 0.008 ± 0.045 | 0.985 |
| CCA-max-IMT change (mm/year) (*n*=552) | 0.009 ± 0.151 | -0.008 ± 0.111 | 0.009 ± 0.118 | 0.875 |
| Mean GSM change(units/year) (*n*=551) | 0.32 ± 7.38 | 0.50 ± 6.08 | 0.61 ± 6.36 | 0.900 |
| Thickened-lesion GSM change (units/year) (*n*=486) | 5.1 ± 12.9 | 6.8 ± 11.6 | 4.5 ± 10.2 | 0.064 |

Continuous variables are presented as means ± standard deviations. Categorical variables are presented as counts (percentages).

*P* for trend was calculated using linear regression models adjusting for age, sex, and each index for atherosclerosis at baseline.

**ESM Table 2. Correlations between Changes in FLP-CGM–derived Metrics and Annual Changes in Index Values of Carotid Atherosclerosis.**

| | Mean glucose change (mmol/l) | CV change (%) | TIR change (%) | TAR$^{>10\ mmol/L}$ change (%) | TBR$^{<3.9\ mmol/L}$ change (%) | $HbA_{1c}$ (%) change (mmol/mol) |
|---|---|---|---|---|---|---|
| Mean IMT change (mm/year) (*n*=522) | 0.055 | -0.048 | -0.037 | 0.050 | -0.045 | 0.067 |
| CCA-max-IMT change (mm/year) (*n*=552) | 0.006 | -0.027 | -0.021 | 0.020 | 0.003 | 0.022 |
| Mean GSM change(units/year) (*n*=551) | -0.032 | 0.009 | 0.012 | -0.018 | 0.018 | -0.013 |
| Thickened-lesion GSM change (units/year) (*n*=486) | 0.064 | 0.016 | -0.046 | 0.053 | -0.029 | 0.032 |

Pearson's correlation coefficients represent the associations between changes in FLP-CGM–derived metrics and annual changes in indexes for carotid atherosclerosis.

**ESM Table 3. Associations between Changes in FLP-CGM–derived Metrics and Annual Changes in IMT.**

| Parameter | Regression coefficient (95% CI) | *P* value |
|---|---|---|
| Mean IMT change | | |
| Mean glucose (1 mmol/l increase) (*n*=494) | 0.002 (-0.001, 0.005) | 0.121 |
| CV (%) (1% increase) (*n*=494) | 0.000 (-0.001, 0.000) | 0.310 |
| TIR change (10% increase) (*n*=494) | 0.001 (0.004, 0.002) | 0.439 |
| $TAR^{>10\ mmol/L}$ (1% increase) (*n*=494) | 0.000 (0.000, 0.000) | 0.171 |
| $TBR^{<3.9\ mmol/L}$ (1% increase) (*n*=494) | -0.001 (-0.001, 0.000) | 0.069 |
| $HbA_{1c}$ (1% increase) (*n*=517) (excluding $HbA_{1c}$) | 0.005 (0.000, 0.011) | 0.072 |
| CCA-max-IMT change | | |
| Mean glucose (1 mmol/l increase) (*n*=494) | 0.001 (-0.006, 0.007) | 0.815 |
| CV (%) (1% increase) (*n*=494) | 0.000 (-0.002, 0.002) | 0.951 |
| TIR change (10% increase) (*n*=494) | -0.002 (-0.008, 0.004) | 0.519 |
| $TAR^{>10\ mmol/L}$ (1% increase) (*n*=494) | 0.000 (0.000, 0.001) | 0.492 |
| $TBR^{<3.9\ mmol/L}$ (1% increase) (*n*=494) | 0.000 (-0.002, 0.002) | 0.844 |
| $HbA_{1c}$ (1% increase) (*n*=517) (excluding $HbA_{1c}$) | 0.006 (-0.010, 0.022) | 0.464 |

The model included age, sex, each IMT value at baseline, body mass index, duration of diabetes, $HbA_{1c}$, systolic blood pressure, total cholesterol, high-density lipoprotein cholesterol, logarithm of triglycerides, serum uric acid, estimated glomerular filtration rate, logarithm of urinary albumin excretion, presence of diabetic retinopathy, smoking status (never smoker, previous smoker, or current smoker), alcohol consumption, use of insulin therapy, use of angiotensin-converting enzyme inhibitors and/or angiotensin II receptor blockers, use of statins, and use of anti-platelet agents

**ESM Table 4. Associations between Changes in FLP-CGM–derived Metrics and Annual Changes in GSM.**

| Parameter | Regression coefficient (95% CI) | *P* value |
|---|---|---|
| Mean GSM change | | |
| Mean glucose change (1 mmol/l increase) (*n*=492) | -0.02 (-0.39, 0.35) | 0.923 |
| CV change (%) (1% increase) (*n*=492) | 0.04 (-0.09, 0.17) | 0.530 |
| TIR change (10% increase) (*n*=492) | -0.14 (-0.49, 0.22) | 0.441 |
| $TAR^{>10\ mmol/L}$ change (1% increase) (*n*=492) | 0.01 (-0.02, 0.04) | 0.579 |
| $TBR^{<3.9\ mmol/L}$ change (1% increase) (*n*=492) | 0.03 (-0.07, 0.13) | 0.588 |
| $HbA_{1c}$ change (1% increase) (*n*=515) (excluding $HbA_{1c}$) | -0.12 (-0.96, 0.72) | 0.772 |
| | | |
| Thickened-lesion GSM change | | |
| Mean glucose change (1 mmol/l increase) (*n*=434) | 0.52 (-0.19, 1.23) | 0.148 |
| CV change (%) (1% increase) (*n*=434) | 0.05 (-0.18, 0.28) | 0.671 |
| TIR change (10% increase) (*n*=434) | -0.45 (-1.12, 0.22) | 0.186 |
| $TAR^{>10\ mmol/L}$ change (1% increase) (*n*=434) | 0.04 (-0.02, 0.11) | 0.195 |
| $TBR^{<3.9\ mmol/L}$ change (1% increase) (*n*=434) | -0.01 (-0.18, 0.17) | 0.952 |
| $HbA_{1c}$ change (1% increase) (*n*=454) (excluding $HbA_{1c}$) | 0.21 (-1.33, 1.76) | 0.786 |

The model included age, sex, each IMT value at baseline, body mass index, duration of diabetes, $HbA_{1c}$, systolic blood pressure, total cholesterol, high-density lipoprotein cholesterol, logarithm of triglycerides, serum uric acid, estimated glomerular filtration rate, logarithm of urinary albumin excretion, presence of diabetic retinopathy, smoking status (never smoker, previous smoker, or current smoker), alcohol consumption, use of insulin therapy, use of angiotensin-converting enzyme inhibitors and/or angiotensin II receptor blockers, use of statins, and use of anti-platelet agents

**ESM Table 5. List of sites and investigators.**

| Site | Investigator |
| --- | --- |
| Ashiya Central Hospital | Koji Matsushita, Manabu Narisawa |
| Aso Clinic | Katsumi Aso, Yuko Ando, Fumihiko Sato |
| Hagiwara Central Hospital | Emiko Morita, Keiichi Torimoto |
| Hayashi Clinic | Isao Hayashi |
| Inokuchi Clinic | Nobuo Inokuchi |
| Japan Community Health Care Organization Osaka Hospital | Yutaka Umayahara, Arichika Deguchi, Azusa Shiraki |
| Juntendo Tokyo Koto Geriatric Medical Center (Department of Medicine, Diabetology, & Endocrinology) | Ayako Kitamura, Eri Tanabe, Hidenori Yoshii, Tomio Onuma, Tomo Nakajima |
| Juntendo University Graduate School of Medicine (Department of Metabolism & Endocrinology) | Eisuke Yasunari, Hideyoshi Kaga, Hiroaki Sato, Hirotaka Watada, Kagemi Takeno, Luka Suzuki, Miwa Himuro, Syuhei Aoyama, Takashi Hunayama, Takehiro Katahira, Takeshi Miyatsuka, Tomoya Mita, Yuya Nishida |
| Juntendo University Nerima Hospital (Department of Medicine, Diabetes, and Endocrinology) | Koji Komiya |
| Kanda Naika Clinic | Satoshi Kawashima |
| Kansai Rosai Hospital (Department of Diabetes and Endocrinology) | Tsunehiko Yamamoto |
| Kihara Diabetes Clinic | Yasuyuki Kihara |
| Kosugi Medical Clinic | Keisuke Kosugi |

| | |
|---|---|
| Kawasaki Hospital (Department of Internal Medicine) | Akihito Otsuka, Jun Murai |
| Matsuoka Medical Clinic | Hirofumi Matsuoka |
| Misaki Naika Clinic | Nobuichi Kuribayashi |
| Japan Labour Health and Safety Organization Kyushu Rosai Hospital, Moji Medical Center (Department of Internal Medicine) | Tadashi Arao, Kei Sugai |
| Musashino Family Clinic | Yuichi Kojima |
| Nakakinen Clinic | Miyoko Saito, Takeshi Osonoi, Yusuke Osonoi |
| Nakama Municipal Hospital | Kohei Uriu, Yosifumi Inada, Kanako Suzuka, Ichiro Takagi |
| National Hospital Organization Osaka National Hospital (Diabetes Center) | Ken Kato |
| Nishida Keiko Diabetes Clinic | Keiko Nishida, Akira Kurozumi, Fumi Uemura, Keiichi Torimoto, Maiko Hajime, Manabu Narisawa, Satomi Sonoda, Kumiko Tidiwa |
| Osaka General Medical Center (Department of Diabetes and Endocrinology) | Youhei Fujita, Sayoko Shimizu, Masahisa Hata, Masahiro Hatazaki |
| Osaka Police Hospital (Department of Endocrinology and Metabolism) | Tetsuyuki Yasuda |
| Osaka Rosai Hospital | Ryomoto Kayoko |
| Osaka University Graduate School of Medicine | Iichiro Shimomura, Naoto Katakami, Takaaki Matasuoka, Mitsuyoshi Takahara, Kazuyuki Miyashita, Hiroyo Ninomiya, Naohiro Taya |

| | |
|---|---|
| (Department of Metabolic Medicine) | |
| Sasaki Hospital | Shinichiro Mine, Kenji Koikawa |
| School of Medicine, University of Occupational and Environmental Health, Japan (First Department of Internal Medicine) | Yosuke Okada, Keiichi Torimoto, Akira Kurozumi, Manabu Narisawa, Maiko Hajime, Fumi Uemura, Satomi Sonoda, Kenichi Tanaka, Takashi Otsuka |
| Secomedic Hospital | Satomi Wakasugi, Tomoya Mita |
| Shiraiwa Medical Clinic | Toshihiko Shiraiwa |
| Taneda Clinic | Yoshinobu Taneda |
| Takaishi Naika Ichoka Clinic | Tomoya Mita |
| Tobata General Hospital (Department of Internal Medicine) | Kazuko Kanda |
| Wakamatsu Hospital of the University of Occupational and Environmental Health | Akira Kurozumi, Nishio Kousuke |
